# Systolic Blood Pressure and Cardiovascular Risk in Patients With Diabetes: A Prospective Cohort Study

**DOI:** 10.1161/HYPERTENSIONAHA.122.20489

**Published:** 2022-12-30

**Authors:** Shishir Rao, Yikuan Li, Milad Nazarzadeh, Dexter Canoy, Mohammad Mamouei, Abdelaali Hassaine, Gholamreza Salimi-Khorshidi, Kazem Rahimi

**Affiliations:** 1 Deep Medicine, Oxford Martin School, University of Oxford, Oxford, United Kingdom (S.R., Y.L., M.N., M.M., G.S.-K., K.R.).; 2 Nuffield Department of Women’s and Reproductive Health, University of Oxford, Oxford, United Kingdom (S.R., Y.L., M.N., M.M., G.S.-K., K.R.).; 3 NIHR Oxford Biomedical Research Centre, Oxford University Hospitals NHS Foundation Trust, Oxford, United Kingdom (K.R.).; 4 Population Health Sciences Institute, University of Newcastle, Newcastle, United Kingdom (D.C.).; 5 Division of Informatics, Imaging and Data Sciences, University of Manchester, Manchester, United Kingdom (A.H.).

**Keywords:** blood pressure, causality, cardiovascular diseases, diabetes mellitus, machine learning

## Abstract

Whether the association between systolic blood pressure (SBP) and risk of cardiovascular disease is monotonic or whether there is a nadir of optimal blood pressure remains controversial. We investigated the association between SBP and cardiovascular events in patients with diabetes across the full spectrum of SBP.

**Methods:**

A cohort of 49 000 individuals with diabetes aged 50 to 90 years between 1990 and 2005 was identified from linked electronic health records in the United Kingdom. Associations between SBP and cardiovascular outcomes (ischemic heart disease, heart failure, stroke, and cardiovascular death) were analyzed using a deep learning approach.

**Results:**

Over a median follow-up of 7.3 years, 16 378 cardiovascular events were observed. The relationship between SBP and cardiovascular events followed a monotonic pattern, with the group with the lowest baseline SBP of <120 mm Hg exhibiting the lowest risk of cardiovascular events. In comparison to the reference group with the lowest SBP (<120 mm Hg), the adjusted risk ratio for cardiovascular disease was 1.03 (95% CI, 0.97–1.10) for SBP between 120 and 129 mm Hg, 1.05 (0.99–1.11) for SBP between 130 and 139 mm Hg, 1.08 (1.01–1.15) for SBP between 140 and 149 mm Hg, 1.12 (1.03–1.20) for SBP between 150 and 159 mm Hg, and 1.19 (1.09–1.28) for SBP ≥160 mm Hg.

**Conclusions:**

Using deep learning modeling, we found a monotonic relationship between SBP and risk of cardiovascular outcomes in patients with diabetes, without evidence of a J-shaped relationship.

**Novelty and Relevance**

**What Is New?**

This study investigated the shape of the association between blood pressure and future risk of cardiovascular outcomes in a cohort of 49 000 patients with diabetes using a validated deep learning causal model (Targeted Bidirectional Electronic Health Records Transformer [T-BEHRT]).

**What Is Relevant?**

In contrast to conventional statistical approaches, the T-BEHRT model showed no evidence of a J-shaped pattern.

Patients in the lowest systolic blood pressure category of <120 mm Hg had the lowest risk of future cardiovascular disease.

**Clinical/Pathophysiological Implications?**

This study extends the lower, the better paradigm of hypertension to patients with diabetes and provides further reassurance about the role of intensive blood pressure lowering in this growing patient population.

Blood pressure (BP) reduction is a well-known primary and secondary preventive strategy for cardiovascular events in addition to diabetes.^1^ Observational studies conducted in low-risk groups have suggested that the association between elevated BP and risk of major cardiovascular disease (CVD) is log-linear.^2,3^ However, the association in patients with pre-existing cardiometabolic disorders is less well understood.

In people with diabetes, reports have been inconsistent in their conclusion about the nature of the association between systolic blood pressure (SBP) and risk of CVD. Although in a cohort of patients with diabetes free of known CVD, the risk of CVD increased continuously with higher SBP, and in the same study when diabetic patients with or without prior CVD were considered, the relationship was J-shaped with the lowest observed risk of cardiovascular events among those with SBP between 130 and 139 mm Hg.^4^ Another study similarly found a J-shaped pattern in patients with diabetes, with the nadir of risk between 135 and 139 mm Hg.^5^ This apparent discontinuous relationship has found some support from conventional meta-analyses of randomized controlled trials. For instance, one study concluded that BP lowering increased the risk of cardiovascular death in trials of patients with diabetes when average SBP was <140 mm Hg at baseline.^6^ However, previous observational studies, which implemented conventional statistical models are prone to issues of reverse causation and uncontrolled confounding,^1,4^ and tabular meta-analyses of trials, which have provided the best level of evidence to date, are vulnerable to the ecological fallacy.^7^

As a consequence, the shape of the association between SBP and cardiovascular endpoints in patients with pre-existing diabetes remains contentious. Recently, better access to rich, longitudinal electronic health records (EHRs) in line with the development of deep learning models for causal inference have provided a new opportunity to overcome issues of uncontrolled confounding that low-dimensional expert-driven statistical approaches are prone to. Methods combining deep learning modeling for rich EHR data utilizing automatic feature extraction and confounding adjustment as well as semiparametric statistical modeling for mitigation of selection biases have shown promising results in several works utilizing semisynthetic and routine clinical EHR data.^8–10^

In this study, we leveraged such novel deep learning methods to re-evaluate the relationship between SBP and cardiovascular events in a sample of 49 000 UK patients with diabetes.

## Methods

### Data Availability

Requests to access the dataset from qualified researchers trained in human subject confidentiality protocols may be sent to the Clinical Practice Research Datalink (CPRD) organization (https://cprd.com/data-access).

### Study Setting and Participants

We used prospectively collected EHR data from CPRD, which has been validated for epidemiological research.^8,11,12^ The CPRD database contains retrospective anonymized patient data covering ≈7% of the UK population and is considered generally representative in terms of sex, age, and ethnicity. The approval for this work was given by the CPRD Independent Scientific Advisory Committee of UK. We used EHR data from 3 resources in which we identified a cohort of 49 000 individuals with prevalent diabetes: primary care, secondary care (Hospital Episode Statistics), and the Office of National Statistics (cause-specific mortality). We included people between 50 and 90 years of age with at least 1 BP measurement taken between the years 1990 and 2005. Consistent with previous studies on BP, the study entry (ie, baseline) was defined as the date of the first BP measurement in this time period.^13–15^ We identified individuals as having diabetes at study entry using validated phenotyping methods; full list of codes for identifying diabetes are available in Table S1.^11,16^ Consistent with previous epidemiological studies, patients with heart failure before study entry were excluded from the study.^1^

The Transparency and Openness Promotion and the Reporting of Studies Conducted Using Observational Routinely-Collected Data reporting guidelines were followed for this cohort study. CPRD Independent Scientific Advisory Committee of UK (protocol: 16_049R) gave the approval for this work.

### Exposures

SBP was the exposure variable and was derived from CPRD measurement data. In CPRD, BP measurement is recorded by general practice staff during an in-person visit or consultation.^12^ The European Society of Cardiology guidelines recommend 3 BP measurements measured 1 to 2 minutes apart with BP recording as the average of the last 2 BP readings.^17^ In CPRD, the general practice staffs follow the same approach but a single BP measurement is recorded from each visit.^12^ We excluded the SBP values <50 and >300 mm Hg as suggested by the previously published phenotyping methods to exclude outlier measurements.^18^ All analyses were conducted with exposure status calculated as mean of SBP measurements in the first 12 months after study entry (ie, baseline exposure period). For example, for a hypothetical individual with 4 measurements in the first 12 months following study entry, the exposure would be the mean value of the 4 measurements. Patients who had a cardiovascular event or left the study during this baseline exposure period were removed from the analyses. Patients were categorized into 6 exposure categories of SBP: <120 mm Hg (reference), 120–129 mm Hg, 130–139 mm Hg, 140–149 mm Hg, 150–159 mm Hg, and ≥160 mm Hg.

### Outcomes

The primary outcome was fatal or nonfatal CVD, defined as a composite of ischemic heart disease (IHD), incident heart failure, stroke, and cardiovascular death. Secondary outcomes were components of the primary outcome: (1) IHD, (2) incident heart failure, and (3) stroke. All outcomes were identified by Read codes (primary care) and *International Classification of Diseases, Tenth Revision* (*ICD-10*) codes (secondary care and mortality data) as reported previously.^11^ Follow-up period started 12 months after study entry; this feature of study design was implemented in order to avoid conducting inference within the exposure period (first 12 months after study entry). Thus, events that occurred between 12 and 120 months after study entry (eg, 108 months or 9 years of follow-up period) were captured for analysis (Figure S1).

### Statistical and Deep Learning Analyses

For the deep learning approach, we used Targeted Bidirectional Electronic Health Records Transformer (T-BEHRT) for risk ratio (RR) estimation of association between SBP and cardiovascular outcomes with SBP of <120 mm Hg considered as reference group.^9^ For each of these comparisons, T-BEHRT was first trained to jointly predict exposure category (propensity score) and risk of outcome with 5-fold cross-validation implemented for training and testing.^19^ The T-BEHRT model incorporated all recorded diagnoses and medications in medical history prior to study entry in addition to baseline smoking status (current, former, and never a smoker)—identified by last known status in the 12 months before baseline—and sex.

Initial estimates were computed on patients from test set of each of the 5 iterations. Second, equipped with these initial estimates, the T-BEHRT model updated risk estimates utilizing doubly-robust post hoc estimator, Cross-Validated Targeted Maximum Likelihood Estimation, to further mitigate biases.^20^ RR and 95% CI were derived from this post hoc estimation procedure. More details and implementation of the T-BEHRT approach can be found in the Supplemental Methods section (Figure S2; Table S2).

To compare against the T-BEHRT, logistic regression (LR) models were implemented to investigate the relationship between SBP and outcomes in individuals with diabetes. The exposure, SBP group was included as a categorical variable. The model was adjusted for age, sex, smoking status at baseline, body mass index (BMI), antihypertensive use at baseline, high-density lipoprotein, total cholesterol, triglycerides, atrial fibrillation, and chronic kidney disease. Baseline BMI, total cholesterol, high-density lipoprotein, and triglycerides were calculated as the average of measurements in the 12 months before baseline. Antihypertensives were identified by British National Formulary codes.^21^ Smoking status (current, former, and never a smoker) was identified by last known status in the 12 months up to baseline. To comply with standard epidemiological research, we conducted imputation before inputting data to LR models. Multiple imputation using chained equations was used to impute missing variables BMI, high-density lipoprotein, total cholesterol, triglycerides, and smoking status; 25 imputations were conducted. For the LR, an estimate for the RR was obtained utilizing direct standardization.^22^ We calculated RR as the average across the test sets of k-fold cross-validation (k=5) and calculated 95% CI over the 5 runs.^23^ Additionally, the crude RR was also calculated as the average risk of the outcome in a particular exposure group divided by the average risk of the same outcome in the reference exposure group.

In addition, to check that findings of our binary regression models were not simply due to unaccounted informed censoring during follow-up, a Cox proportional hazards model was implemented with the same predictors as for the LR model. As is done traditionally for Cox proportional hazards modeling, censoring and time of cardiovascular event data were used for the modeling. The proportional hazards assumption was tested by plotting Schoenfeld residuals.

As a sensitivity analysis, to check that the impact of more complete adjustment of covariates, we extended our adjustment of the LR model with the following additional variables: insulin use at baseline, mean of measured values of hemoglobin A1c in the 12 months up to study entry, and duration of diabetes at baseline. As a measurement, hemoglobin A1c measurements were missing for 45.1% of patients in our cohort. Multiple imputation using chained equations was used to impute missing variables hemoglobin A1c, BMI, high-density lipoprotein, total cholesterol, triglycerides, and smoking status; 25 imputations were conducted.

Several additional sensitivity analyses were conducted using the T-BEHRT model. First, we conducted analyses stratified by sex (male and female) and age (≤75 and 75 years of age) analyses. Second, since antihypertensives can dilute associations, we repeated our main analysis with patients who had not taken antihypertensives after study entry.^5^ Third, we conducted stratified analysis of the primary outcome additionally by baseline antihypertensive use. Fourth, to assess the possible impact of reverse causation, we excluded individuals who had cardiovascular events in the first 12 and 24 months of the follow-up period (24 and 36 months after study entry). Fifth, we repeated analysis without excluding patients who had an event in the first 12 months after study entry.

All data processing, data imputation, and analyses were undertaken in the programming language, Python. The programming code for the T-BEHRT model is found as an open-source package on the Deep Medicine research group codebase (https://github.com/deepmedicine). Please see the Major Resources Table in the Supplemental Material.

## Results

### Population Statistics

A total of 49 000 patients with diabetes were included in this study (Figure S3). Descriptive statistics of the cohort according to the baseline SBP are shown in Table 1. Median age at baseline was 65 years of age. Forty-five percent of the individuals in the cohort were women and 39% current or former smokers. Patients in lower SBP exposure groups had higher prevalence of baseline IHD and lower proportion of antihypertensive use. BMI at baseline generally indicated an overweight cohort across all exposure groups. Prior to index date, patients had a median of 10 (interquartile range, 4–22) number of visits to general practice or hospital (Table S3). The median follow-up duration was 7.3 years. A total of 16 378 (33.4%) patients had cardiovascular events in the follow-up period, and 12 797 (26.1%) patients were censored (ie, patients who died due to causes other than CVD and who were otherwise lost to follow-up; Table 1; Figure S3). Event rates are provided in the Supplemental Material (Table S4).

**Table 1.**
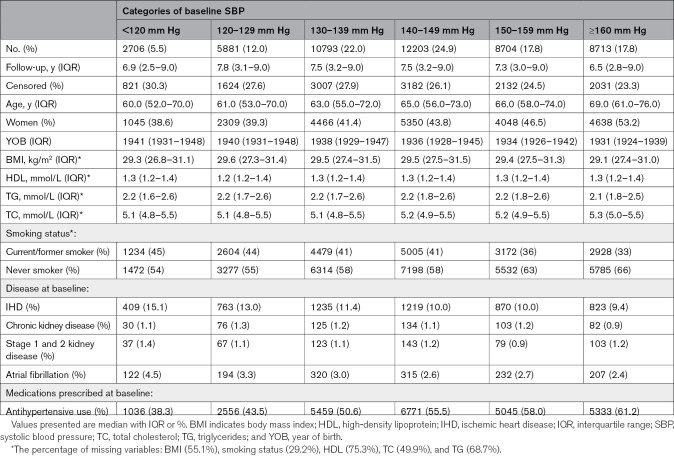
Descriptive Statistics for Patients in Each Systolic Blood Exposure Group

### Association of SBP on Cardiovascular Events

**Figure 1.**
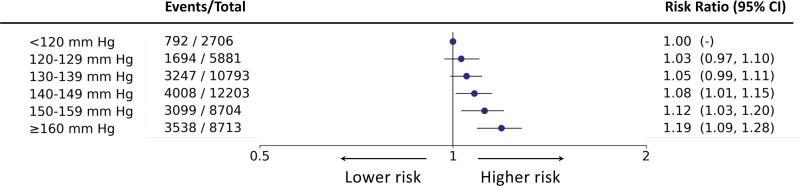
**Forest plot of risk ratio estimates of the Targeted Bidirectional Electronic Health Records Transformer (T-BEHRT) model with 95% CI for association of systolic blood pressure (SBP) and the primary composite outcome.** From the left, the 6 exposure groups are shown in first column. Number of events and total number of patients in each exposure group is shown in second column. The forest plot and corresponding risk ratio estimates are shown in the right-most column relative to reference class, <120 mm Hg. The forest plot is plotted in logarithmic scale. For all estimates for reference class, there is no CI.

**Figure 2.**
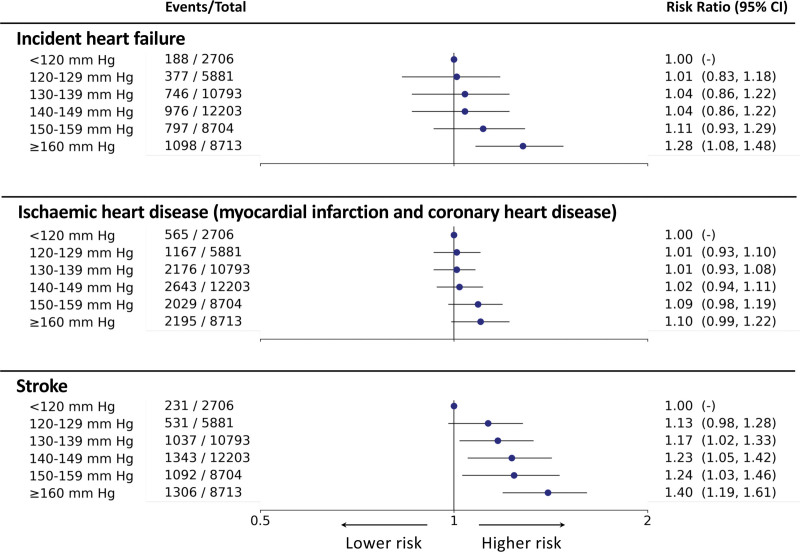
**Forest plot of risk ratio estimates of the Targeted Bidirectional Electronic Health Records Transformer (T-BEHRT) model with 95% CI for association of systolic blood pressure (SBP) and secondary outcomes.** From the left, the 6 exposure groups are shown in first column. Number of events and total number of patients in each exposure group is shown in second column. The forest plot and corresponding risk ratio estimates are shown in the right-most column relative to reference class, <120 mm Hg. The forest plot is plotted in logarithmic scale. For all estimates for reference class, there is no CI.

RR estimates from adjusted T-BEHRT model demonstrated a rise in the risk of cardiovascular events with a rise in SBP categories (Figure 1). Estimates investigating the association between SBP and secondary outcomes showed that the lowest risk of all secondary cardiovascular outcomes was observed at <120 mm Hg (Figure 2).

**Figure 3.**
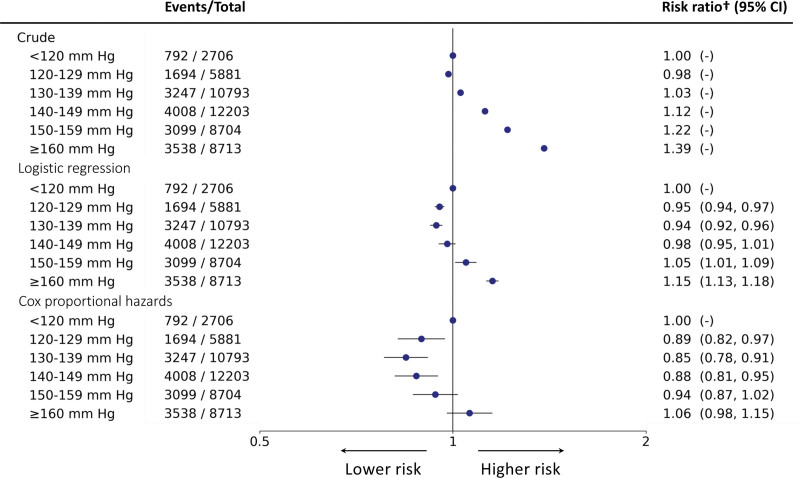
**Forest plot of relative risk estimates of various conventional statistical models with 95% CI for association of systolic blood pressure (SBP) and the primary outcome.** From the left, the 6 exposure groups are shown in first column. Number of events and total number of patients in each exposure group is shown in second column. The forest plot and corresponding risk ratio (hazard ratio for Cox proportional hazards model) estimates are shown in the right-most column relative to reference class, <120 mm Hg. The forest plot is plotted in logarithmic scale. For all crude estimates and estimates for reference class, there is no CI. The † indicates hazard ratio for the Cox proportional hazards model.

By contrast, the crude, LR, and the Cox proportional hazards models depicted a J-shaped pattern with all models capturing a nadir of risk at SBP between 130 and 139 mm Hg (Figure 3). These patterns were largely driven by the J-shaped associations of SBP with risk of incident heart failure and IHD (Figure S4). However, for stroke, the crude and adjusted LR models estimated that <120 mm Hg SBP demonstrated lowest risk similar to the T-BEHRT model. The adjusted Cox proportional hazards approach found that SBP between 130 and 139 mm Hg exhibited the lowest risk of all secondary outcome investigations. In sensitivity analysis of primary and secondary outcomes using an LR model with additional adjustment variables, the trends were almost identical to those captured by the main adjusted LR approach (Table S5).

**Figure 4.**
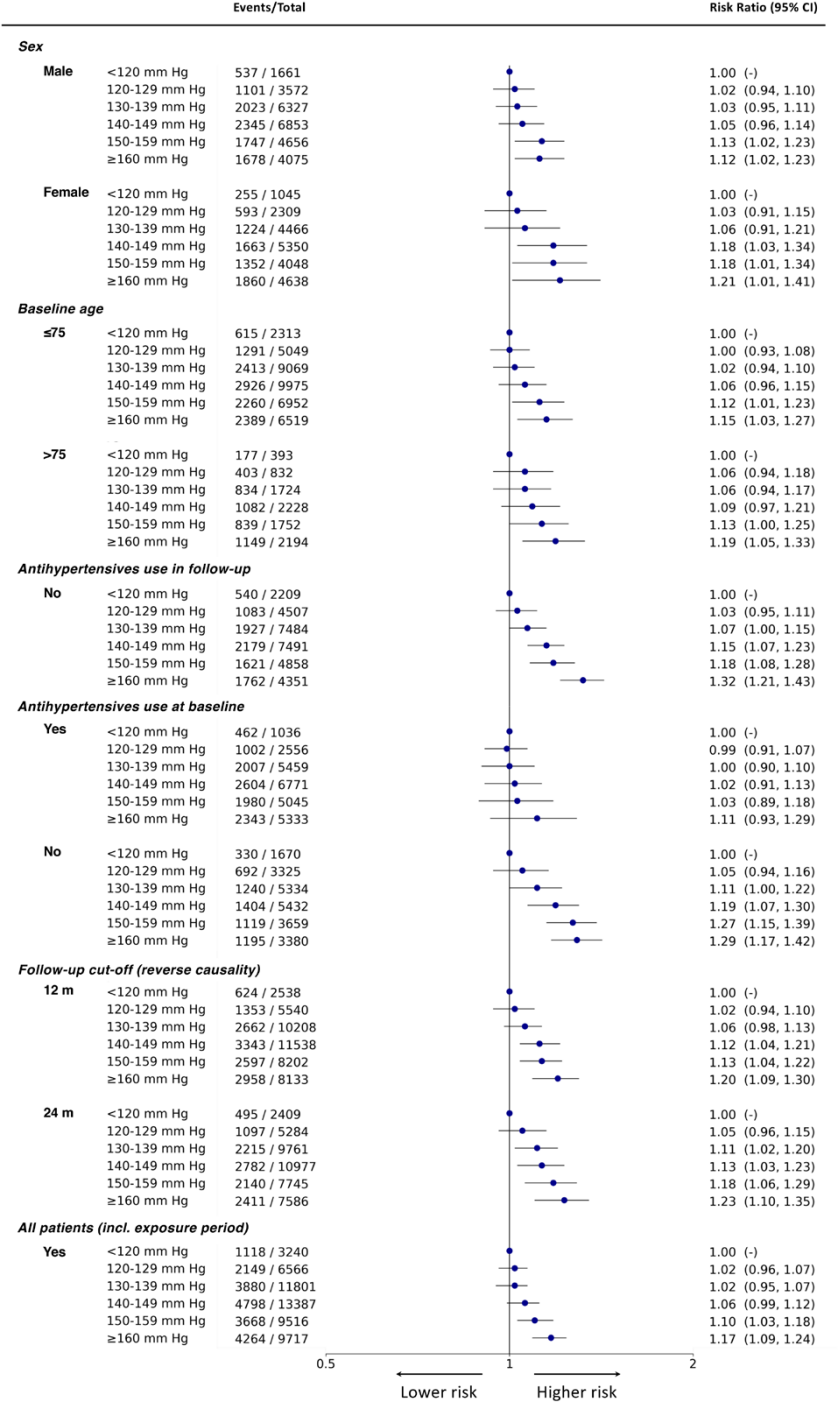
**Forest plot of risk ratio estimates of the Targeted Bidirectional Electronic Health Records Transformer (T-BEHRT) model with 95% CI for association of systolic blood pressure (SBP) and the primary outcome in sensitivity analyses.** The particular sensitivity analysis is italicized on the left with strata indented. From the left, the 6 exposure groups are shown in the first column. Number of events and total number of patients in each exposure group is shown in the second column. The forest plot and corresponding risk ratio estimates are shown in the right-most column relative to reference class, <120 mm Hg. The forest plot is plotted in logarithmic scale. For all estimates for reference class, there is no CI.

Shown in Figure 4, the sensitivity analyses investigating the association between SBP and the primary composite outcome using the T-BEHRT model preserved the trend found in the main analysis. In both sex and age-stratified analyses, the log-linear trend across the spectrum of SBP was generally preserved. In analysis of patients who had not taken antihypertensives during the exposure and follow-up periods, the RR estimates and corresponding 95% CI for each exposure group was slightly higher than their counterparts in the main analysis but overall mirrored the trend in the main analyses. Additionally, stratifying by antihypertensive usage at baseline, albeit the slight presence of a local minimum, the trend showed little material difference from the main result. Furthermore, excluding patients who had events in the first 12 and 24 months of follow-up also captured a similar trend as that of the main analysis. Lastly, incorporating those who had dropped out during the first 12 months following baseline (ie, the exposure period), the trend presented was similar to that of the main analysis.

## Discussion

In this study, using deep learning modeling on a comprehensive dataset of UK EHR in a cohort of 49 000 individuals with diabetes, we found SBP to be monotonically associated to cardiovascular risk. Patients with SBP <120 mm Hg exhibited the lowest risk of cardiovascular outcomes. These results were compared with conventional approaches in epidemiology and were robust to several sensitivity analyses.

Findings from previous observational studies investigating the association of SBP and cardiovascular events in patients with diabetes utilizing conventional statistical modeling have shed light on a larger phenomenon: the shape of the association as a function of the comprehensiveness of confounding adjustment. Solely adjusting for confounders such as baseline age, sex, and demographic variables fell short of eliminating the J-shaped association in cohorts with diabetes free of CVDs as shown in past studies such as the Swedish National Diabetes Registry study with 54 000 patients and the ROSE study (Retrospective Epidemiological Study to Investigate Outcome and Mortality with Glucose Lowering Drug Treatment in Primary Care) with 34 000 patients.^24,25^ However, extending the predictor set to include key cardiovascular risk factors more comprehensively modeled the association as compared to the former 2 studies and thus rejected the J-shaped association in a cohort of 187 000 patients.^4^ However, in the same study, including patients with pre-existing cardiovascular conditions, the conventional modeling consistently captured the J-shaped relationship for all outcomes, with the exception of stroke.^4^ Similarly, our analysis using LR modeling with adjustment for several commonly used confounders exhibited evidence of the J-shaped relationship between SBP and risk of compositive CVD, incident heart failure, and IHD. More extensive adjustment or use of a survival model did not change the shape of the association.

Conventional statistical models are usually implemented in observational studies with curated, low-risk cohorts relatively free of multimorbidity at baseline. By ensuring the cohort is healthy, statistical models may only require a handful of established confounding variables to sufficiently provide adjusted and trustworthy estimates. However, in cohorts like ours with high baseline BMI and a host of underlying conditions—including some cardiovascular in nature indicating prevalent multimorbidity—conventional approaches with expert selected confounders and regression modeling might be insufficient for capturing complex interactions and confounding.

Our work implementing deep causal modeling addresses these issues and fills some of the gaps in this research of SBP and cardiovascular outcomes. With T-BEHRT modeling, our research found no evidence of the J-shaped association between SBP and primary and secondary outcomes. While a recent individual-participant data meta-analysis of randomized evidence has indirectly dismissed the existence of a J-shaped in patients with or without prior CVD,^1^ evidence of heterogeneity of treatment effects in patients with diabetes has been controversial. In particular, a tabular meta-analysis of randomized trials in patients with diabetes has suggested that BP lowering might increase the risk of cardiovascular death when SBP is <140 mm Hg. While not directly analyzing BP lowering, our observational study, on the contrary, provides evidence that SBP maintains a log-linear relationship with cardiovascular outcomes across a wide range of baseline SBP categories in patients with diabetes.

We note in our cohort of high-risk patients, relatively modest associations were observed using the T-BEHRT approach. This, however, was also the case in our conventional modeling and comparable with previous research.^4^ The weak associations might be due to 2 main reasons. First, as recently shown by trial evidence, the relative effect of BP lowering on cardiovascular outcomes was half as strong in people with diabetes as those without diabetes.^26^ This might be due to the fact that part of the effect of BP lowering on CVD outcomes is mediated through prevention of diabetes; a pathway that might not be relevant to people with pre-existing diabetes.^27^ Second, our cohort included patients with several comorbidities and use of several medications. While all these attributes are typical in diabetes patients (and a suitable case for complex models such as T-BEHRT), they could lead to attenuation of true associations with CVD as compared to associations in lower risk cohorts.^4,28^ This is supported by our sensitivity analyses, where exclusion of patients with use of antihypertensives at baseline or during follow-up led to stronger relationship between SBP and risk of CVD.

These results have important implications for cardiovascular research. Our analysis provides some clarity concerning the relationship between SBP and cardiovascular events in patients with diabetes. Hence, while our investigation on its own is insufficient for recommending revisions of hypertension guidelines, our work rather functions as independent analyses complementing the findings of the individualized patient data meta-analysis of randomized evidences.^26^ Together, they support the lower the better paradigm of SBP in patients with diabetes.

### Strengths and Limitations

Our first strength is CPRD allows us access to a host of rich diagnosis, medication, and measurements variables. Also, we leveraged phenotyping methods validated for CPRD. Unlike previous studies of SBP and cardiovascular events that have excluded older individuals, patients between 50 and 90 years of age were included in this work. Exclusion based on baseline attributes was limited; only those with heart failure were excluded at baseline.

Second, deep learning feature extraction was used to automatically adjust for confounding variables and latent interactions in our input data. We also implemented conventional statistical approaches enabling direct comparison of T-BEHRT to established methods in the study of SBP and cardiovascular outcomes. We showed that by using superior adjustment methods, we can more effectively model the association in observational data, thereby rejecting the J-shaped argument.

This study also has several limitations. EHR data might have some level of measurement error or misclassification. Despite endorsement of the validity and reliability of physician diagnoses in the CPRD dataset, especially concerning studies of cardiovascular disorders,^29^ case ascertainment of diabetes might have misclassifications (eg, metabolic syndromes and related disorders as opposed to diabetes). Measurement errors are a natural issue with EHR data, especially that of BP, but we have attempted to mitigate these issues in the case of SBP data by taking an average of multiple measurements over the course of 12 months following baseline. However, we are unable to make any direct inference about the relationship in particular patient groups such as those with white coat hypertension.

T-BEHRT framework typically requires high-dimensional longitudinal data. The T-BEHRT estimator can function optimally with access to multiple EHR modalities (diagnoses, medications, etc) and associated temporal annotation (age and calendar year). Furthermore, as previously reported, when such rich data are provided, the unsupervised learning component (masked EHR modeling) of the T-BEHRT model works well in reducing bias in estimation.^9^ When data are sparse or limited (finite sample), the doubly-robust estimation, with known benefits for finite-sample estimation, more accurately estimates RR than the variants without utilization of doubly-robust estimation.^20^ Furthermore, when positivity (overlap) between exposure groups is limited, the T-BEHRT model fares better than other benchmark models.^22^ Specifically, in our study of patients with cardiometabolic multimorbidity, where the traditional LR model insufficiently captures confounder relationships, deep learning modeling in tandem with semiparametric methods can be appropriately implemented for robust RR estimation. Further testing to assess model estimation accuracy is needed for settings in which the exposure is poorly defined (eg, generic painkiller) or when the outcome is categorical or continuous. Future confirmatory investigations on additional data sources would be invaluable for studying the association from different perspectives.

Our deep learning approach functions in the binary outcome framework as opposed to the time-to-event framework, which would be a more optimal framework for analyses where time-dependent bias is likely. Specifically, failure to carefully consider censoring in cohort can bias downstream estimation of the association. However, even though deep learning for survival analyses has been explored in the risk prediction setting, methodological issues in estimation of hazard ratios using nonlinear, deep learning models have limited pursuits of survival modeling for population-level association estimation. Hence, methodological studies would be necessary to develop and demonstrate the utility of deep learning-driven survival models for EHR, which could be applied to investigate the studied association and other challenging questions. Lastly, as is the case for all observational studies, our model is unable to overcome the challenge of fully taking account of unmeasured confounding and when this is of concern, alternative study designs such as randomized controlled trial might be required.

### Perspectives

Using large-scale, linked EHR, our investigation utilizing a deep learning model concluded that patients with diabetes in the lowest category of SBP of <120 mm Hg exhibited the lowest cardiovascular risk. This was in contrast to expert-dependent conventional approaches that have largely defended the paradoxical nonlinear J-shaped association between SBP and cardiovascular end points in those with diabetes. This difference is likely explained by the deep learning model’s ability to more comprehensively capture known and latent confounders in routine EHR, thus reducing the potential of important confounders being missed out. In light of a recent meta-analysis of trials demonstrating that BP lowering medications reduces the risk of cardiovascular events in patients with diabetes regardless of baseline BP, our investigation provides complementary evidence against a causal link between low BP and risk of CVD in this population. Together these lines of evidence support the lower, the better paradigm of BP management. In terms of methodological insights, our work demonstrates that where complex confounding might be at play, conventional modeling is more likely to fall short of delivering well-adjusted estimates of risk. In such scenarios, models such as T-BEHRT, which are capable of automatically capturing known and latent confounding in minimally processed, rich EHR provide a worthwhile alternative for minimizing biases. Future research applying and testing such data-driven deep learning methods in high-risk cohorts would be beneficial for disentangling the nuances of risk and protection in patients with pre-existing conditions.

## Article Information

### Author Contributions

S. Rao and K. Rahimi conceived the study concept and designed the study. K. Rahimi and S. Rao acquired and curated the data. S. Rao conducted the data analysis and analysis visualizations. M. Nazarzadeh contributed to data analysis. S. Rao wrote the initial draft of the article. K. Rahimi is the guarantor for the study. All of the authors contributed to the revisions of the article and had full access to the data.

### Sources of Funding

This work was supported by grants from the British Heart Foundation ([BHF]; grant number, FS/PhD/21/29110 to Y. Li and K. Rahimi), (grant number, FS/19/36/34346 to M. Nazarzadeh and K. Rahimi), and (grant number, PG/18/65/33872 to K. Rahimi and D. Canoy); United Kingdom Research and Innovation (UKRI) Global Challenges Research Fund (GCRF; grant number, ES/P0110551/1 to K. Rahimi); Oxford National Institute of Health Research (NIHR) Biomedical Research Center (to K. Rahimi), Novo Nordisk grant (to M. Mamouei), and the Oxford Martin School (OMS), University of Oxford (to K. Rahimi). The funders had no role in study design, data collection and analysis, decision to publish, or preparation of the article. The views expressed are those of the authors and not necessarily those of the OMS, the BHF, the GCRF, the NIHR, or the Department of Health and Social Care.

### Disclosures

None.

### Supplemental Material

Supplemental Methods

Tables S1–S5

Figures S1–S4

Major Resources Table

## Supplementary Material
