# Supplementary material for: Systolic Blood Pressure and Cardiovascular Risk in Patients With Diabetes: A Prospective Cohort Study
Source: Hypertension. 2022 Dec 30;80(3):598–607. doi: 10.1161/HYPERTENSIONAHA.122.20489 (PMC9944753; doi:10.1161/HYPERTENSIONAHA.122.20489)
Supplement: Supplementary file 2 [file hyp-80-598-s002.docx]

# Supplementary Material

Systolic blood pressure and cardiovascular risk in patients with diabetes: a prospective cohort study

Shishir Rao, MSc^1,2^, Yikuan Li, MSc^1,2^, Milad Nazarzadeh, MSc^1,2^, Dexter Canoy, PhD^4^, Mohammad Mamouei, PhD^1,2^, Abdelaali Hassaine, PhD^5^, Gholamreza Salimi-Khorshidi, PhD^1,2^, Kazem Rahimi, DM, FESC^1,2,3 *^

^1^ Deep Medicine, Oxford Martin School, University of Oxford, Oxford, United Kingdom

^2^ Nuffield Department of Women’s & Reproductive Health, University of Oxford, Oxford, United Kingdom

^3^ NIHR Oxford Biomedical Research Centre, Oxford University Hospitals NHS Foundation Trust, Oxford, United Kingdom

^4^ Population Health Sciences Institute, University of Newcastle, Newcastle, United Kingdom

^5^ Division of Informatics, Imaging & Data Sciences, University of Manchester, Manchester, United Kingdom

**Supplementary Methods**

Study design for the investigated association between systolic blood pressure (SBP) and cardiovascular endpoints is visualised in **Supplementary Figure S1.** The study entry date, (i.e., baseline) denoted as “SE” in the figure, which is the first date of SBP measurement between ages 50 and 90 and years 1990 and 2005, the exposure period where repeat SBP measurements are averaged to serve as exposure status (12 months), and the follow-up period (108 months), which starts 12 months after index date are shown in this diagram.

For the deep learning approach, we used Targeted Bidirectional Electronic Health Records Transformer (T-BEHRT) for causal estimation of association between SBP and cardiovascular outcomes^1^. The model, T-BEHRT, utilises an extended BEHRT architecture (**Supplementary Figure S2A**) to model both longitudinal variables (e.g., diagnoses/medications) with attributes of age and calendar year of recording and static variables (e.g., sex) native to EHR data to conduct initial estimation of RR ^2^. BEHRT is a deep learning model for EHR that has achieved state-of-the-art performance on numerous EHR based machine learning prediction tasks including incident heart failure prediction ^2,3^.

For inclusion of EHR variables in the T-BEHRT model, we conducted data processing of raw CPRD data. First, diagnostic codes were harmonized by mapping Read codes from primary care and ICD-10 codes from hospital data to a total of 1,497 unique diagnostic codes. Second, we mapped CPRD medication codes in the “Product code” format to 386 codes in the BNF coding format ^4^. Third, we derived the variable, smoking status (current, former, never a smoker) identified by last known status in the 12 months before baseline. Fourth and finally, patient sex was extracted from CPRD for modelling.

Each longitudinal variable (e.g., disease) is represented by an embedding matrix ^2^, which is a two-dimensional matrix with each instance as a vector (**Supplementary Figure S2B)**. Age and calendar are made into categorical variables and also represented by embedding matrices. The encounter (disease/medication) embedding and the corresponding embeddings for age and calendar year of recording were summed and used as a model predictor. The static variables – sex and smoking status at baseline, which are binary and categorical variables respectively – were also embedded as vectors.

Additionally, T-BEHRT leverages powerful unsupervised learning in tandem with the Masked EHR Modelling (MEM) to learn better latent representations that predict treatment (i.e., propensity score estimation) ^1,5^. The intuition: by learning better features that predict exposure – i.e., pre-exposure variables associated with exposure, the model automatically learns important features, a “subset” of which are confounders ^5^. By extracting and adjusting for these known and latent confounding variables more robustly than other conventional models in statistics and more recent deep learning approaches, the model achieves state-of-the-art finite sample RR estimation demonstrated in a host of experiments ^1^.

CV-TMLE is the post-hoc estimator used to update initial estimation of RR^6^. We conducted propensity score trimming and excluded patients with predicted propensity score greater than 0.97 and less than 0.03 before pursuing RR calculation ^1^.

For implementation of the T-BEHRT model, logistic regression, CV-TMLE, and imputation methods, we used the python coding language. For deep learning, two NVIDIA Titan Xp Graphical Processing Units (GPU) were used. For statistical imputation and modelling, the sklearn package was used. Lastly hyperparameters for the model – i.e., non-trainable parameters of the model are shown in **Supplementary Table S2**; number of encounters considered before baseline is 200, the hidden size was 150, and the weight for the end-to-end unsupervised learning objective (MEM) was 0.1. Further details of the T-BEHRT model and implementation can be found in the original methods publication^1^.

**Supplemental References**

1. Rao S, Mamouei M, Salimi-Khorshidi G, Li Y, Ramakrishnan R, Hassaine A, Canoy D, Rahimi K. Targeted-BEHRT: Deep Learning for Observational Causal Inference on Longitudinal Electronic Health Records. *IEEE Trans Neural Netw Learn Syst* 2022; 1–12.

2. Li Y, Rao S, Solares JRA, Hassaine A, Ramakrishnan R, Canoy D, Zhu Y, Rahimi K, Salimi-Khorshidi G. BEHRT: Transformer for Electronic Health Records. *Sci Rep* 2020; 10: 7155.

3. Rao S, Li Y, Ramakrishnan R, Hassaine A, Canoy D, Cleland JG, Lukasiewicz T, Salimi-Khorshidi G, Rahimi K. An explainable Transformer-based deep learning model for the prediction of incident heart failure. *IEEE J Biomed Health Inform* 2022; 1–1.

4. Trowell WJ. “British National Formulary.” *British Medical Journal (Clinical research ed.)*. Epub ahead of print 1981. DOI: 10.1136/bmj.282.6269.1078.

5. Rosenbaum PR, Rubin DB. The central role of the propensity score in observational studies for causal effects. *Biometrika*. Epub ahead of print 1983. DOI: 10.1093/biomet/70.1.41.

6. Levy J. An Easy Implementation of CV-TMLE. *arXiv*.

**Supplemental Tables and supporting information**

**Supplementary Table S1. Codes for identifying diabetes**

| **Type** | **Setting (coding system)** | **Codes** |
| --- | --- | --- |
| **Diagnoses** | Primary (medcode) | 506, 711, 758, 1038, 1323, 1407, 1549, 1647, 1682, 1684, 2340, 2342, 2471, 2475, 2478, 2986, 3286, 3837, 4513, 5002, 5884, 6509, 7059, 7069, 7328, 7563, 7795, 8414, 8618, 8836, 8842, 9013, 9881, 10098, 10099, 10418, 10659, 10692, 10755, 10977, 11094, 11551, 11599, 11626, 11663, 11677, 11848, 11930, 12213, 12262, 12307, 12455, 12506, 12507, 12640, 12675, 12682, 12703, 12736, 13069, 13070, 13074, 13078, 13196, 13279, 14803, 14889, 15690, 16230, 16490, 16491, 16502, 16881, 17067, 17247, 17262, 17313, 17545, 17817, 17858, 17859, 17869, 18056, 18142, 18143, 18167, 18209, 18219, 18230, 18264, 18278, 18311, 18387, 18390, 18425, 18496, 18505, 18642, 18683, 18747, 18766, 18777, 19739, 20696, 21472, 21482, 21689, 21983, 22487, 22573, 22871, 22884, 22967, 23479, 24327, 24363, 24423, 24490, 24571, 24693, 24694, 24836, 25591, 25636, 26054, 26108, 26604, 26605, 26855, 27891, 27921, 28574, 28622, 28769, 28856, 29041, 29979, 30294, 30323, 30477, 31053, 31310, 31790, 32193, 32403, 32556, 32627, 33254, 33343, 33807, 33969, 34152, 34268, 34283, 34450, 34528, 34912, 35105, 35107, 35321, 35383, 35385, 35399, 35785, 36633, 36695, 36798, 37315, 37648, 37806, 38076, 38078, 38130, 38161, 38617, 38986, 39070, 39317, 39420, 39809, 40023, 40401, 40682, 40837, 40962, 41049, 41389, 41686, 41716, 42505, 42567, 42729, 42762, 42831, 43139, 43227, 43453, 43493, 43785, 43857, 43921, 43951, 44033, 44260, 44440, 44443, 44779, 44982, 45250, 45276, 45467, 45491, 45499, 45919, 46150, 46290, 46301, 46521, 46533, 46577, 46624, 46917, 46963, 47011, 47032, 47058, 47321, 47370, 47377, 47409, 47582, 47584, 47649, 47650, 47816, 47954, 48078, 48192, 49074, 49146, 49276, 49554, 49655, 49869, 49884, 49949, 50175, 50225, 50429, 50527, 50609, 50813, 50960, 50972, 51261, 51697, 51756, 51957, 52104, 52212, 52236, 52237, 52283, 52303, 53200, 53238, 53392, 53634, 54008, 54212, 54600, 54601, 54856, 54899, 55075, 55123, 55239, 55431, 55842, 56268, 56448, 56803, 57278, 57333, 57389, 57621, 57723, 58133, 58159, 58604, 58639, 59253, 59288, 59365, 59725, 59903, 59991, 60107, 60208, 60499, 60699, 60796, 61021, 61071, 61122, 61344, 61523, 61670, 61829, 62107, 62146, 62209, 62352, 62613, 62674, 63017, 63357, 63371, 63412, 63690, 63762, 64142, 64283, 64357, 64446, 64449, 64571, 64668, 65025, 65062, 65267, 65463, 65616, 65704, 66145, 66274, 66675, 66872, 66965, 67212, 67664, 67853, 67905, 68105, 68390, 68792, 68818, 68843, 69124, 69152, 69278, 69676, 69748, 69993, 70316, 70448, 70766, 70821, 72320, 72345, 83485, 85991, 90301, 91646, 91942, 91943, 93380, 93390, 93468, 93491, 93529, 93631, 93657, 93704, 93727, 93854, 93870, 93875, 93878, 93922, 94011, 94186, 94383, 94699, 94955, 94956, 95093, 95094, 95159, 95343, 95351, 95539, 95553, 95636, 95992, 95994, 96010, 96235, 96506, 97281, 97446, 97474, 97809, 97824, 97849, 97894, 98071, 98392, 98616, 98704, 98723 |
|  | Secondary (International classification of diseases -10^th^ revision) | O24.3, O24.2, O24.1, O24.0, N08.3, M14.2, H36.0, H28.0, G63.2, G59.0, E10-E14 |
| **Medications** | Primary (British National Formulary) | Chapters 6.1.1 and 6.1.2 |

**Supplementary Table S2.** **Hyperparameters for the T-BEHRT model**

| **Hyperparameter** | **Attribute** |
| --- | --- |
| Mini-batch size | 128 |
| Hidden size of BEHRT | 150 |
| Intermediate BEHRT Layer size | 108 |
| Hidden dropout probability | 0.3 |
| Attention dropout probability | 0.4 |
| Number of hidden layers (BEHRT) | 4 |
| Hidden activation functions | Gaussian error linear unit (GeLU) |
| Initialiser range of parameters | 0.02 |
| *N (number of encounters utilised before baseline)* | 200 |
| *d (weight for unsupervised learning objective)* | 0.1 |

**Supplementary Table S3. Statistics for visits at baseline stratified by exposure group**

|  | Number of visits | |
| --- | --- | --- |
|  | Median | IQR |
| <120 mm Hg | 10 | 4,25 |
| 120-129 mm Hg | 10 | 4,23 |
| 130-139 mm Hg | 9 | 4,22 |
| 140-149 mm Hg | 9 | 4,22 |
| 150-159 mm Hg | 9 | 4,22 |
| ≥160 mm Hg | 10 | 4,21 |

IQR: interquartile range

**Supplementary Table S4. Primary and secondary outcome events per 1000 patient-years stratified by exposure group**

|  | Primary outcome | HF | IHD | Stroke |
| --- | --- | --- | --- | --- |
| <120 mm Hg | 49.9 | 10.7 | 34.5 | 13.3 |
| 120-129 mm Hg | 46.6 | 9.3 | 31.0 | 13.3 |
| 130-139 mm Hg | 48.7 | 10.1 | 31.4 | 14.2 |
| 140-149 mm Hg | 53.3 | 11.6 | 33.5 | 16.2 |
| 150-159 mm Hg | 58.5 | 13.4 | 36.6 | 18.6 |
| ≥160 mm Hg | 69.7 | 19.1 | 40.5 | 23.1 |

IHD: ischemic heart disease; HF: heart failure

**Supplementary Table S5. Risk ratio estimation for primary and secondary cardiovascular endpoints with adjusted logistic regression modelling (extended adjustment set)**

| Outcome | Primary  (RR; 95% CI) | Incident HF  (RR; 95% CI) | IHD  (RR; 95% CI) | Stroke  (RR; 95% CI) |
| --- | --- | --- | --- | --- |
| <120 mm Hg | 1.0 | 1.0 | 1.0 | 1.0 |
| 120-129 mm Hg | 0.95; (0.94, 0.97) | 0.91; (0.85, 0.98) | 0.92; (0.88, 0.95) | 1.04; (0.99, 1.1) |
| 130-139 mm Hg | 0.95; (0.93, 0.96) | 0.88; (0.83, 0.94) | 0.89; (0.85, 0.93) | 1.06; (0.99, 1.12) |
| 140-149 mm Hg | 0.99; (0.95, 1.02) | 0.99; (0.91, 1.06) | 0.90; (0.85, 0.96) | 1.16; (1.13, 1.18) |
| 150-159 mm Hg | 1.06; (1.01, 1.10) | 1.08; (0.99, 1.18) | 0.99; (0.92, 1.05) | 1.29; (1.24, 1.35) |
| ≥160 mm Hg | 1.15; (1.13, 1.18) | 1.41; (1.28, 1.54) | 1.04; (0.99, 1.09) | 1.47; (1.35, 1.6) |

RR: risk ratio; 95% CI: 95% confidence interval; HF: heart failure; IHD: ischaemic heart disease

**Supplemental Figures and Figure Legends**


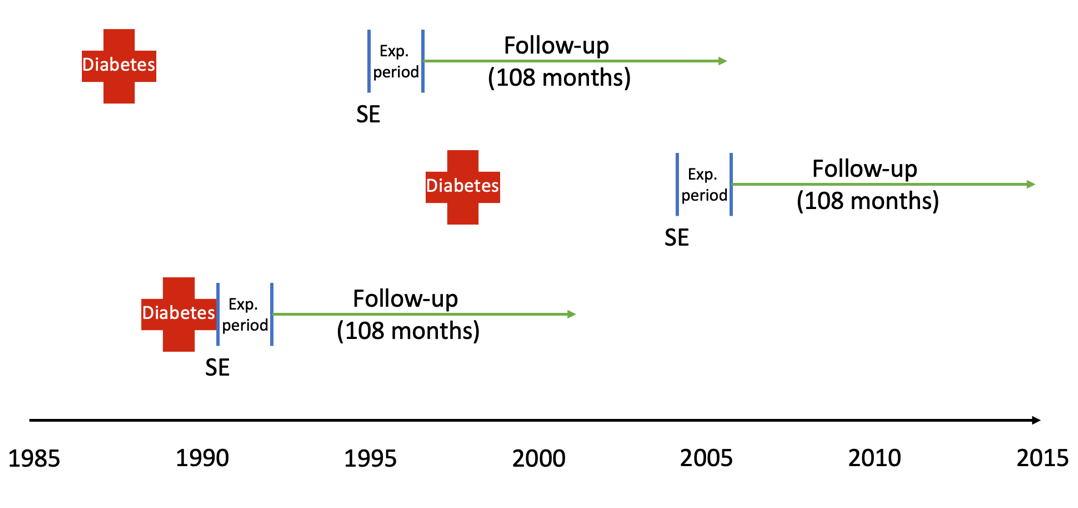


Supplementary Figure S1. Study design of the association between systolic blood pressure (SBP) and cardiovascular endpoints in those with diabetes for three hypothetical patients. Study entry (SE) (i.e., baseline) for a patient is the date of the first SBP measurement recorded between 1990 and 2005 between ages 50 and 90. “Exp. period” denotes the exposure period (12 months) and the follow-up of 108 months is denoted with the green arrow. Diabetes status at baseline is captured through validated phenotyping; the time of diabetes before baseline varies across patients. The x-axis is calendar year ranging from 1985 to 2015.


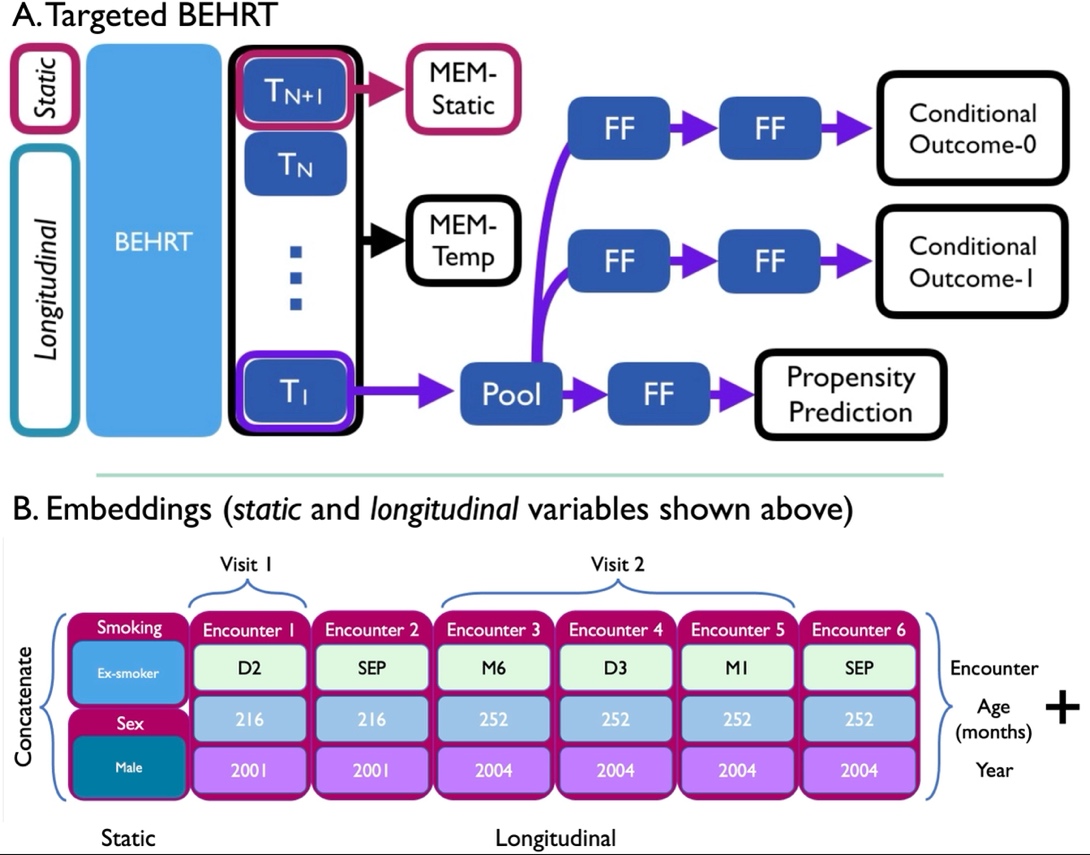


Supplementary Figure S2: T-BEHRT model architecture (A) and data embedding design (B). In (A), the T-BEHRT model is shown with the static and longitudinal input, BEHRT feature extractor, latent outputs T_1_ to T_N+1_ (one for each diagnosis/medication and static variables inputted), and four tasks for causal estimation. 1. MEM unsupervised training to better extract latent confounders, 2. Conditional outcome prediction 0 – the prediction of the outcome given the individual has received treatment 0 (i.e., reference group), 3. Conditional outcome prediction 1 – the prediction of the outcome given the individual has received treatment 1 (i.e., not reference group), 4. Propensity score prediction (prediction of treatment status). In (B), we show how the model handles longitudinal and static input. The diagnoses (e.g. hypothetical D2, D3) and medications (e.g., M1, M6) are supplemented with attributions of time – both relative in terms of age at time of record (e.g., 216 months of age) and absolute in terms of calendar year (e.g. 216). The “SEP” input informs the model of a new visit. The embeddings for encounter, age, and year are summed to form a predictor embedding (“+” sign in the right). The static variables smoking status and sex are concatenated and inputted (shown in left). T-BEHRT: Targeted BEHRT; EHR: electronic health records; SEP: Separator; MEM: Masked EHR Modelling; FF: feed forward / multi-layer perceptron neural network


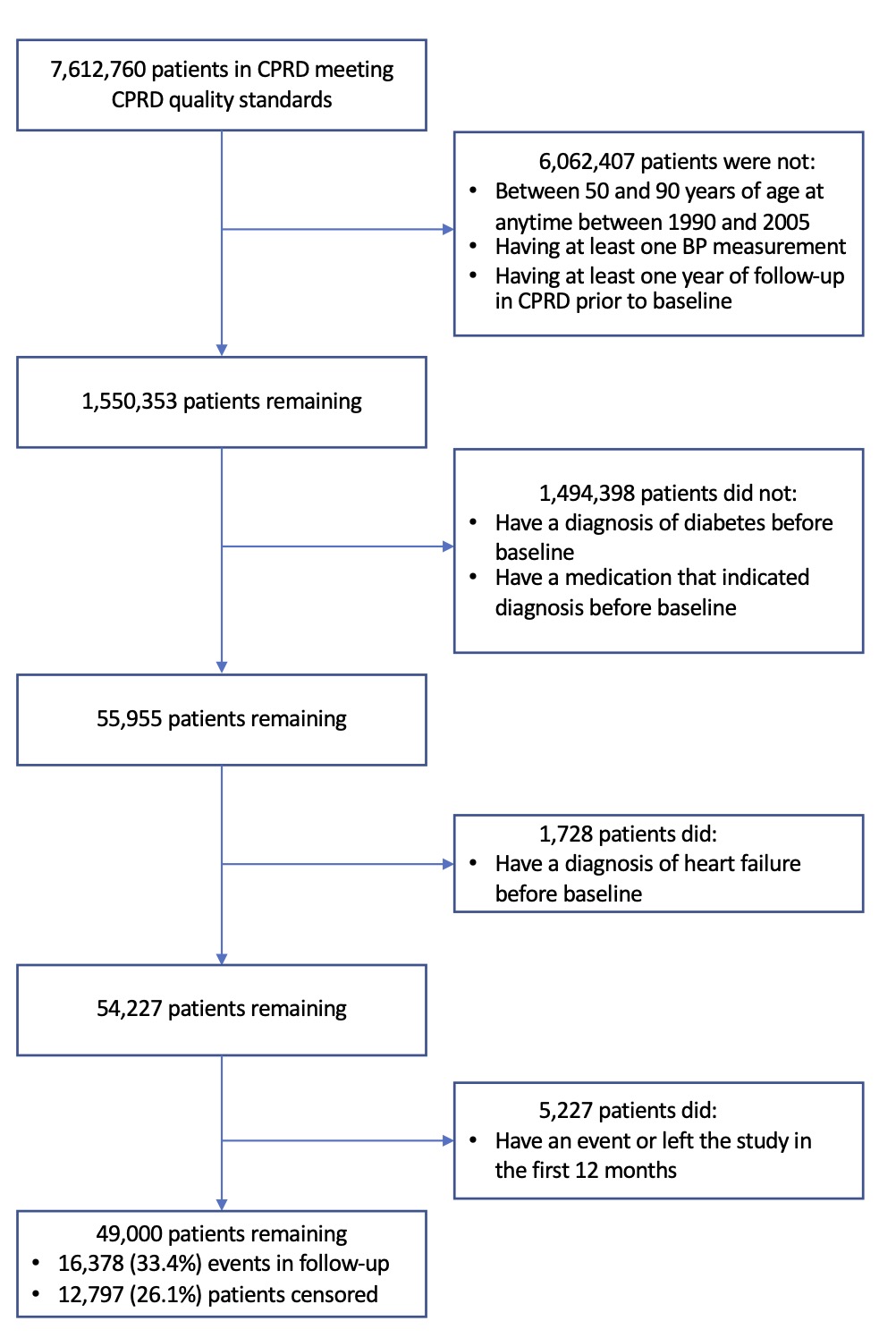


Supplementary Figure S3: Flowchart figure for cohort selection procedures


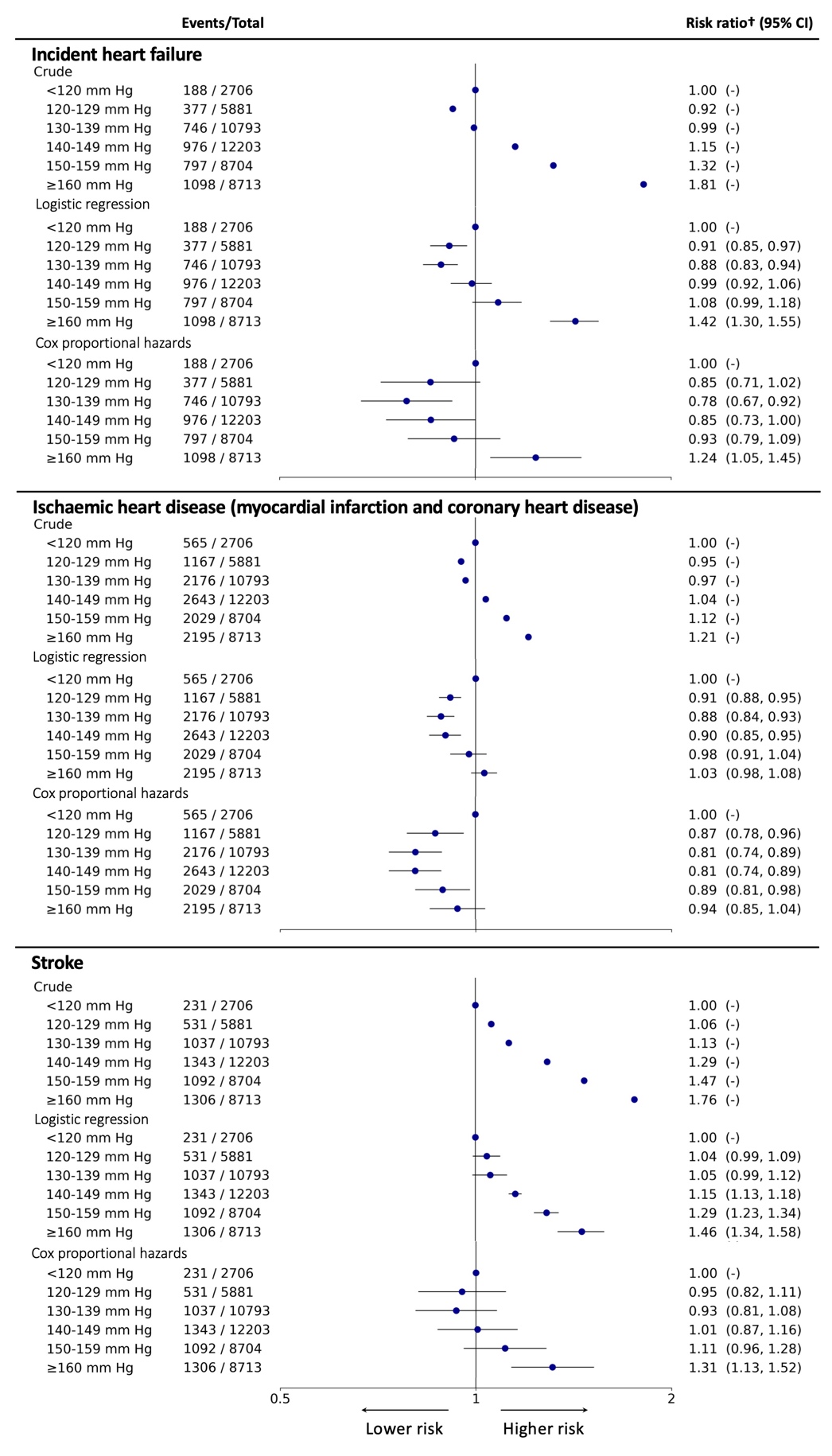


Supplementary Figure S4: Forest plot of relative risk estimates of various conventional statistical models with 95% confidence intervals (CI) for association of systolic blood pressure and secondary outcomes. From the left, the six exposure groups are shown in first column. Number of events and total number of patients in each exposure group is shown in second column. The forest plot and corresponding risk ratio (hazard ratio for Cox proportional hazards model) estimates are shown in the right-most column relative to reference class, <120 mm Hg. The forest plot is plotted in logarithmic scale. For all crude estimates and estimates for reference class, there is no confidence interval. †: hazard ratio for the Cox proportional hazards model.

**Supplemental Major Resources Table**

| **Description** | **Source / Repository** | **Persistent ID / URL** |
| --- | --- | --- |
| Clinical Practice Research Datalink (CPRD) | Data upon requesting the CPRD organization | https://cprd.com/data-access |
| Code for Targeted-BEHRT | GitHub Codebase | https://github.com/deepmedicine/Targeted-BEHRT |
